# Virulence Characteristics of *mecA*-Positive Multidrug-Resistant Clinical Coagulase-Negative Staphylococci

**DOI:** 10.3390/microorganisms8050659

**Published:** 2020-05-01

**Authors:** Jung-Whan Chon, Un Jung Lee, Ryan Bensen, Stephanie West, Angel Paredes, Jinhee Lim, Saeed Khan, Mark E. Hart, K. Scott Phillips, Kidon Sung

**Affiliations:** 1 Division of Microbiology, National Center for Toxicological Research, US Food and Drug Administration, Jefferson, AR 72079, USA; alvarmar@naver.com (J.-W.C.); Saeed.Khan@fda.hhs.gov (S.K.); mark.hart@fda.hhs.gov (M.E.H.); 2 Division of Cardiology, Albert Einstein College of Medicine, Bronx, NY 10461, USA; unjung.lee@einsteinmed.org; 3 Department of Chemistry and Biochemistry, University of Oklahoma, Norman, OK 73019, USA; rcbensen@ou.edu; 4 Department of Animal Science, University of Arkansas, Fayetteville, AR 72701, USA; stephaniewest2012@gmail.com; 5 NCTR-ORA Nanotechnology Core Facility, US Food and Drug Administration, Jefferson, AR 72079, USA; Angel.Paredes@fda.hhs.gov (A.P.); jihee3140@gmail.com (J.L.); 6 Department of Microbiology and Immunology, University of Arkansas for Medical Sciences, Little Rock, AR 72205, USA; 7 Division of Biology, Chemistry, and Materials Science, Office of Science and Engineering Laboratories, Center for Devices and Radiological Health, US Food and Drug Administration, Silver Spring, MD 20993, USA; Kenneth.Phillips@fda.hhs.gov

**Keywords:** coagulase-negative staphylococci, multidrug-resistant, virulence

## Abstract

Coagulase-negative staphylococci (CoNS) are an important group of opportunistic pathogenic microorganisms that cause infections in hospital settings and are generally resistant to many antimicrobial agents. We report on phenotypic and genotypic virulence characteristics of a select group of clinical, *mecA*-positive (encoding penicillin-binding protein 2a) CoNS isolates. All CoNS were resistant to two or more antimicrobials with *S. epidermidis* strain 214EP, showing resistance to fifteen of the sixteen antimicrobial agents tested. Aminoglycoside-resistance genes were the ones most commonly detected. The presence of megaplasmids containing both horizontal gene transfer and antimicrobial resistance genetic determinants indicates that CoNS may disseminate antibiotic resistance to other bacteria. *Staphylococcus sciuri* species produced six virulence enzymes, including a DNase, gelatinase, lipase, phosphatase, and protease that are suspected to degrade tissues into nutrients for bacterial growth and contribute to the pathogenicity of CoNS. The PCR assay for the detection of biofilm-associated genes found the *eno* (encoding laminin-binding protein) gene in all isolates. Measurement of their biofilm-forming ability and Spearman’s rank correlation coefficient analyses revealed that the results of crystal violet (CV) and extracellular polymeric substances (EPS) assays were significantly correlated (ρ = 0.9153, P = 3.612e-12). The presence of virulence factors, biofilm-formation capability, extracellular enzymes, multidrug resistance, and gene transfer markers in *mecA*-positive CoNS clinical strains used in this study makes them powerful opportunistic pathogens. The study also warrants a careful evaluation of nosocomial infections caused by CoNS and may be useful in studying the mechanism of virulence and factors associated with their pathogenicity in vivo and developing effective strategies for mitigation.

## 1. Introduction

While coagulase-negative staphylococci (CoNS) live as normal microflora of skin and mucous membranes in humans and animals, they also are increasingly recognized as important pathogenic bacteria that cause hospital-acquired infections [1,2]. They have been associated with bacteremia, bloodstream infections, bone and joint infections, endocarditis, osteomyelitis, urinary tract infections, and wound infections. CoNS are especially a threat to immunocompromised patients, such as those with anticancer therapy, intensive care, premature birth, and transplant, and patients with implanted foreign body materials [3]. Distinguishing pathogenic and contaminating commensal isolates remains a major challenge in clinical practice because various CoNS have different capabilities to cause infection [1].

Formation of biofilm plays a pivotal role in the virulence of CoNS by allowing cells to persist in the human body and evade the host immune defense system [4]. Therefore, biofilm formation may be a useful indicator to measure the virulence characteristics of pathogenic CoNS. Biofilms contain polysaccharide intercellular adhesion (PIA) and biofilm-associated protein (Bap) [5]. PIA is synthesized by N-acetylglucosaminyltransferase encoded by the *icaADBC* gene cluster. Other cell surface-associated proteins, including bone sialoprotein binding protein (*bbp*), clumping factors A and B (*clfA*, *clfB*), collagen binding protein (*cna*), elastin binding protein (*ebpS*), fibronectin binding proteins A and B (*finbA*, *finbB*), and laminin binding protein (*eno*), also contribute to biofilm formation [6]. CoNS are observed to form biofilms on or around a variety of medical devices, such as central venous catheters, prosthetic heart valves, and pacemakers [7]. Biofilm protects bacteria residing inside of the structure against antibiotics used to treat infections [8]. Furthermore, reduced metabolic activity due to slow growth of bacterial cells inside the biofilm matrix lowers uptake of antibiotics. Therefore, cells in a biofilm can develop a high level of antibiotic resistance that reaches up to one thousand-fold that of planktonic cells [8]. Resistance to antimicrobial agents has a close association with pathogenicity mechanisms in CoNS [9]. Not only are CoNS resistant to multiple antibiotics, like β-lactams, aminoglycosides, and macrolides that are currently used for treatment of methicillin-resistant *S. aureus* infections, but they also can be a reservoir for transmission of antimicrobial resistance genes to other pathogenic bacteria [4]. The prevalence of methicillin-resistant CoNS has been reported in many sources, such as the hospital environment, nares of healthy humans, outpatients, bacteremia, and bloodstream infections [10,11]. Methicillin-resistant CoNS carry a *mecA* gene, which codes for the membrane-bound penicillin-binding protein 2a (PBP 2a), and can be disseminated by horizontal transfer from one staphylococcal strain to another [12]. Barbier and coworkers reported that a high prevalence of *mecA*-positive CoNS might increase the transmission of *mecA* to *S. aureus* strains, promoting the emergence of new methicillin-resistant *S. aureus* clones [13]. Additionally, the high frequency of methicillin-resistant CoNS has increased the use of glycopeptide antibiotics like vancomycin and teicoplanin [14].

CoNS produce other virulence factors, including deoxyribonuclease (DNase), gelatinase, lipase, proteases, and toxins [1]. By producing DNase, CoNS are able to degrade extracellular DNA, thereby avoiding the immune response [15]. The lipase of staphylococci has been known to inactivate bactericidal lipids and assist bacterial survival in human skin [16]. The same enzyme may resist attacks by phagocytes and granulocytes and play a key role in biofilm formation. Proteases inactivate elastin, immunoglobulins (IgA, IgG, and IgM), plasma proteinase inhibitors, and tissue adhesion molecules [17]. Gelatinase, a zinc metalloprotease, can hydrolyze gelatin, collagen, casein and other proteins; it is involved in biofilm production and is responsible for endocarditis [18].

Whereas there have been many studies regarding virulence characteristics of methicillin-resistant *S. aureus*, limited studies have been reported on CoNS [19,20]. Therefore, investigation of phenotypic and genotypic virulence characteristics in clinical CoNS isolates is of great value for understanding their roles in pathogenesis. In this study we investigated antibiotic resistance, virulence factors, and biofilm formation characteristics of CoNS obtained from human clinical samples.

## 2. Materials and Methods

### 2.1. Bacterial Strains 

Twenty-nine *mecA*-positive clinical CoNS isolated from various sources, including nasal, catheter, blood, urine, perirectal, and wound, during January 2010 to October 2011 were used in this study (Table 1). The bacterial isolates were grown at 37 °C overnight in brain heart infusion broth (BHI, BD, Franklin Lakes, NJ, USA) or on trypticase soy agar (TSA) containing 5% sheep blood (BD) depending on the experimental needs.

### 2.2. Antimicrobial Susceptibility

Assays for antibiotic susceptibility were performed using the Kirby–Bauer disk-diffusion method [21]. The antibiotics used for disk diffusion assays included ampicillin (AMP, 25 μg), bacitracin (BAC, 10 units), cefazolin (CEF, 30 μg), ciprofloxacin (CIP, 5 μg), erythromycin (ERY, 15 μg), gentamicin (GEN, 30 μg), kanamycin (KAN, 30 μg), lincomycin (LIN, 2 μg), novobiocin (NOV, 30 μg), oxacillin (OXA, 1 μg), penicillin (PEN, 10 units), polymyxin B (POL, 300 units), rifampicin (RIF, 5 μg), streptomycin (STR, 10 μg), tetracycline (TET, 30 μg), and vancomycin (VAN, 30 μg). Antibiotic discs were purchased from Thermo Fisher Scientific (Wilmington, DE, USA). Zones of inhibition were measured after growth of bacteria overnight at 37 °C on Mueller–Hinton (MH, BD) agar plates, and according to CLSI guidelines the susceptibility of test isolates to antimicrobials was interpreted [22]. *S. aureus* ATCC 25,923 was used as a quality control.

### 2.3. Chromosomal and Plasmid DNA Isolation

CoNS were grown at 37 °C overnight in BHI broth and chromosomal DNA was extracted by using a QIAamp DNA Mini Kit (Qiagen, Valencia, CA, USA), following the supplier’s instructions. A modified alkaline lysis method was used to extract plasmid DNA [23]. An overnight broth culture was centrifuged and the pellet was mixed with alkaline lysis solution (20 mM Tris-HCl (pH 7.0), 50 mM EDTA (pH 8.0), 0.58 M sucrose) and lysostaphin (1 mg/mL) (Sigma-Aldrich Co., St. Louis, MO, USA). Then, the mixture was incubated at 37 °C for 30 min. The second lysis buffer (0.1 M NaOH, 1% sodium dodecyl sulfate (SDS)) was added and incubated on ice for 5 min. After that, 1.5 M potassium acetate (pH 4.8) was added to the mixture and incubated on ice once again for 5 min. The lysate was centrifuged and the supernatant was mixed with phenol:chloroform:isoamyl alcohol (25:24:1). Plasmid DNA in the aqueous layer was precipitated with cold 100% ethanol at −80 °C, washed with 70% ethanol, and dissolved with TE buffer (10 mM Tris-HCl (pH 7.5), 1 mM EDTA (pH 8.0)). After DNA extraction, each 1.5 µL of the DNA sample was loaded on top of the sensor of a Nanodrop 2000 UV spectrophotometer (Thermo Fisher Scientific) and DNA purity was measured by the absorbance at 260 and 280 nm. DNA profiles were revealed by 0.8% agarose gel electrophoresis at 100 V for 4 h and a supercoiled DNA from Agilent Technologies (Santa Clara, CA, USA) was employed as a molecular size marker.

### 2.4. Amplification of Antibiotic-Resistance, Horizontal Transfer, and Pathogenicity-Associated Genes by PCR

Antibiotic resistance, horizontal transfer and virulence-associated genes were detected by primers that were previously reported [24,25,26,27,28,29,30,31,32,33,34,35,36] (Supplementary Tables S1–S3). PCR reactions were carried out in 50 µL total volume containing 25 μL Thermo Scientific DreamTaq PCR master mix (2×) (Thermo Scientific, Oakwood Village, OH, USA), 2 µL of 10 µM forward and reverse primer mix, and 10 ng of genomic DNA extract. PCR cycling conditions were as follows—initial denaturation for 3 min at 95 °C, 35 cycles of denaturation for 30 sec at 95 °C, annealing for 30 sec at an appropriate temperature depending on melting temperature of the primers, extension for 1 min at 72 °C, and final extension for 15 min at 72 °C. PCR amplicons were run on a 1.0% agarose gel stained with 1x GelRed (Biotium, Inc., Fremont, CA, USA), and visualized using a Molecular Imager^®^ Gel Doc™ XR System (BIO-RAD, Hercules, CA, USA). Presumptive positive PCR products or bands were purified by using a QIAquick Gel Extraction kit (Qiagen) and sequenced by Retrogen, Inc. (San Diego, CA, USA). Each sequenced amplicon was aligned and analyzed using DNASTAR Lasergene software package v. 12 (Madison, WI, USA).

### 2.5. Tests for Virulence Factors and Invasion

Tests for the production of coagulase were performed using rabbit plasma (Hardy Diagnostics, Santa Maria, CA, USA) [37]. CoNS were grown in BHI broth at 37 °C for 20 h and then 50 µL of bacterial suspension was mixed with rabbit plasma. Then, clotting of the rabbit plasma was checked after incubation at 37 °C for 4 h. Toluidine blue broth (Hardy Diagnostics), containing 0.01% toluidine blue O as a color indicator and casein and soy peptones as nutrient sources, was utilized for the detection of DNase. A suspension of CoNS at 4.0 McFarland standard was inoculated into the broth and a color interpretation was read following 6 h incubation [38]. In a gelatinase test, a fresh CoNS culture of high cell density was stab-inoculated deep into 12% nutrient gelatin (Hardy Diagnostics) and incubated at 25 °C for up to 7 days. Gelatinase-positive strains showed hydrolysis of gelatin when the tubes were incubated in an ice bath for 30 min [39]. For detection of lipase activity, bacteria were incubated for 72 h at 37 °C on spirit blue agar (BD) supplemented with lipase reagent, tributyrin and polysorbate 80 [40]. Lipase-positive isolates produced colonies with clear halos. Casein agar plates (Thermo Scientific Remel, Lenexa, KS, USA) containing instant nonfat dry milk were used for testing protease activity [38]. Inoculated CoNS on casein agar were cultured at 35 °C for up to 21 days to confirm clear halo zones around colonies. Phosphatase and urease tests were done by the VITEK 2 automated system (bioMerieux, Hazelwood, MO, USA) according to the manufacturer’s recommendations.

### 2.6. Biofilm Formation Test by CV, EPS and MTT Methods

For the crystal violet (CV) biofilm formation assay [41], an overnight-grown culture of CoNS was diluted with fresh tryptic soy broth (TSB) (BD) media in 96-well microplates (Sigma-Aldrich Co.). After incubation of bacteria in static conditions for 20 h at 37 °C, the microplates were washed three times with filter-sterilized water and stained with a 0.1% solution of crystal violet (CV) (Sigma-Aldrich Co.). Then, the microplates were washed with filter-sterilized water and air-dried. The CV bound to the biofilm was solubilized with a 30% solution of glacial acetic acid (Sigma-Aldrich Co.) and the optical density was determined at 550 nm using a Synergy 2 Multi-mode microplate reader (BioTek Instruments, Inc., Winooski, VT, USA). To compare biofilm-forming ability, a 3-(4,5-dimethyl-2-thiazolyl)-2,5-diphenyl-2H-tetrazolium bromide (MTT) (Sigma-Aldrich Co.) reduction assay was employed [42]. As in the CV method, MTT was used for staining. Following solubilization of MTT-stained material by dimethyl sulfoxide (DMSO) (Sigma-Aldrich Co.), the absorbance was measured at 570 nm. To measure the production of extracellular polymeric substances (EPS), TSB and Congo red (0.5 mM) (Sigma-Aldrich Co.) were added to the biofilm, which was washed with phosphate-buffered saline (PBS) (Sigma-Aldrich Co.) [43]. After incubation for 2 h at 37 °C, the medium was centrifuged at 10,000 rpm for 5 min and the optical density of the supernatant was read at 490 nm. All biofilm experiments employed the same inoculum concentration (OD_600_ = 0.1) and were performed in triplicate. *S. epidermidis* RP62A and *S. aureus* ATCC 25,923 were used for positive and negative controls, respectively.

Statistical data were analyzed by R statistical software [44]. The ANOVA assumptions were tested for the data of the CV, EPS and MTT assays. The Anderson–Darling Goodness of Fit test was conducted to determine normality. Since the null hypothesis was rejected at the 0.05 significance level, nonparametric analysis was used to compare the assays and explore the relationship of methods with each isolate. Spearman rank correlation coefficients were calculated to determine associations among the different methods of biofilm formation testing and their relationships were described by principal component analysis (PCA). The confidence level for significance in all tests was 95%.

### 2.7. Field Emission Scanning Electron Microscopy (FESEM) and Negative-Stain Transmission Electron Microscopy (NS-TEM)

A CoNS biofilm was grown on Thermanox polyester coverslips (Thermo Fisher Scientific) in BHI broth overnight at 37 °C. The coverslips were rinsed three times with PBS for 15 min and dehydrated using 15%, 30%, 50%, 70%, 90%, and 95% ethanol for 20 min and absolute (100%) ethanol for 30 min at room temperature. The samples were dried in an Autosamdri-815, Series A automatic critical point drier (Tousimis Research Corporation, Rockville, MD, USA) using liquid CO_2_ and sputter-coated with gold (Denton Vacuum, Moorestown, NJ, USA). Images then were visualized using a Zeiss-Merlin FESEM (Carl Zeiss Microscopy, Thornwood, NY, USA). For NS-TEM, overnight-grown biofilm cells were washed with PBS. A drop of the biofilm cells was placed onto a formvar/carbon-coated nickel grid (Polysciences, Warrington, PA, USA) and washed with PBS. Negative staining was carried out using 2% uranyl acetate and the samples were observed with a JEOL 2100 TEM (Peabody, MA, USA).

## 3. Results

### 3.1. Phenotypic and Genotypic Antimicrobial Resistance

**Figure 1:**
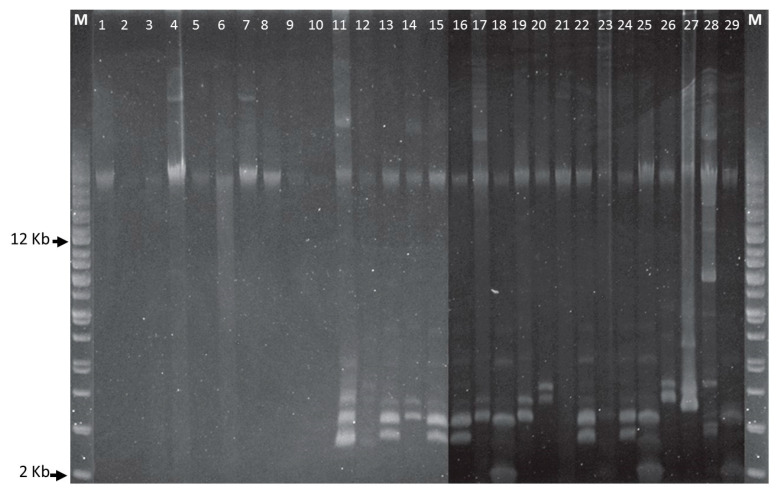
Plasmid DNA profiles of *Staphylococcus* species. Agarose gel electrophoresis (0.8%) was run at 100 V for 4 h and a supercoiled DNA ladder from Agilent Technologies was used as a molecular size marker. First and last lanes are supercoiled plasmid DNA ladders. *Staphylococcus* strains in lanes 1-29—10SC, 20SC, 30SC, 40SC, 50SC, 60SC, 70SC, 80SC, 91HA, 101HA, 111HA, 121HA, 132LE, 143AU, 153AU, 163AU, 174EP, 184EP, 194EP, 204EP, 214EP, 224EP, 235HO, 245HO, 255HO, 266LU, 276LU, 287SI, and 297SI.

All CoNS isolates were multidrug resistant and all *S. haemolyticus* isolates showed resistance to at least ten or more antibiotics (Table 1). Almost ninety percent (26/29) of the CoNS were resistant to PEN. Antimicrobial resistance for OXA, AMP, BAC, ERY, CIP, LIN, and STR were 75.9%, 72.4%, 69%, 58.6%, 55.2%, 55.2%, and 48.3%, respectively, but VAN resistance was found only in the *S. epidermidis* 174EP isolate (Table 1). *S. epidermidis* strain 214EP showed resistance against the largest number (13/16) of antimicrobial agents. Among aminoglycoside antibiotic resistance determinants, the most dominant gene was *aac(6′)-Ie-aph(2′)-Ia* (93.1%)*,* followed by *aph(3′)-IIIa* (58.6%)*, aac(6′)-aph(2″)* (44.8%), and *ant(4′)-Ia* (20.7%). Only two *S. epidermidis* isolates, 174EP and 194EP, had no aminoglycoside resistance determinants. *tetK* and *tetL* were detected at low rates between 10–20%. The *str*, *tetM*, *tetS*, *tetW*, and class I integron genes were not found in any isolate. Although most *S. sciuri* isolates carried only megaplasmids, other CoNS strains carried multiple plasmids with various sizes (Figure 1). Regarding horizontal transfer genes, the *pre* relaxase genes of the staphylococcal mobilizable plasmids pT181 and pSK41 were found in 62.1% (18/29) and 48.3% (14/29), respectively. The *traL* gene was detected in only two isolates, *S. sciuri* strain 60SC and *S. haemolyticus* strain 121HA. All CoNS were negative for PCR amplification using primers designed to amplify the other transfer genes, including the *nes* relaxase genes of pSK41, *traE*, *traG*, *traK*, and *traM*.

### 3.2. Phenotypic and Genotypic Virulence Factors in Clinical CoNS Isolates

DNase activity was observed in most isolates (27/29) except *S. lentus* strain 132LE and *S. hominis* strain 235HO (Table 2). Gelatinase and protease were found in most *S. sciuri* strains. Urease activity was found in *S. epidermidis*, *S. hominis*, *S. lugdunensis* and *S. simulans*. All CoNS species were shown to be positive for lipase by splitting tributyrin and polysorbate 80, except for *S. haemolyticus*.

Genes for fibrinogen-binding protein (*clfA*) and intracellular adhesion protein (*icaA*) were detected by PCR at higher percentage (40–50%) than other virulence genes, such as *fnbB*, *fib*, *bap*, *clfB*, *icaB*, and *icaD* (Table 3). Enolase (*eno*), encoding a laminin-binding protein, was present in all CoNS, but other virulence genes, including collagen-binding protein (*cna*), elastin-binding protein (*ebpS*), fibronectin-binding protein (*fnbA*), bone sialoprotein-binding protein (*bbp*)*,* hemolysins (*hla*, *hlb*), staphylococcal enterotoxins (*sea*, *seb*, *sec*, *sed*, *see*, *seg*, *seh*, *sei*, *sej*), toxic shock syndrome toxin (*Tst*), exfoliative toxins (*eta*, *etb*), and Panton–Valentine leukocidin (*pvl*), were not present in any isolate.

### 3.3. Biofilm Formation

**Figure 2:**
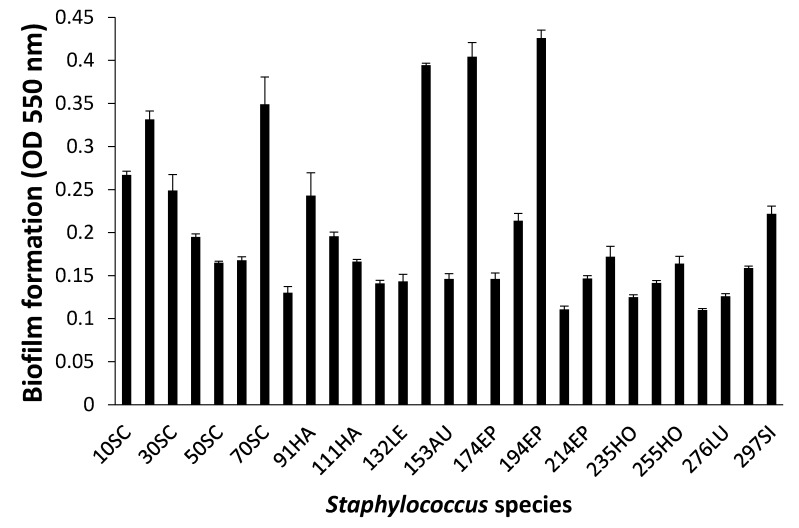
Biofilm formation of *Staphylococcus* species shown by crystal violet (CV) staining. Experiments were run in triplicate and each bar represents the mean ± standard deviation from the mean.

**Figure 3:**
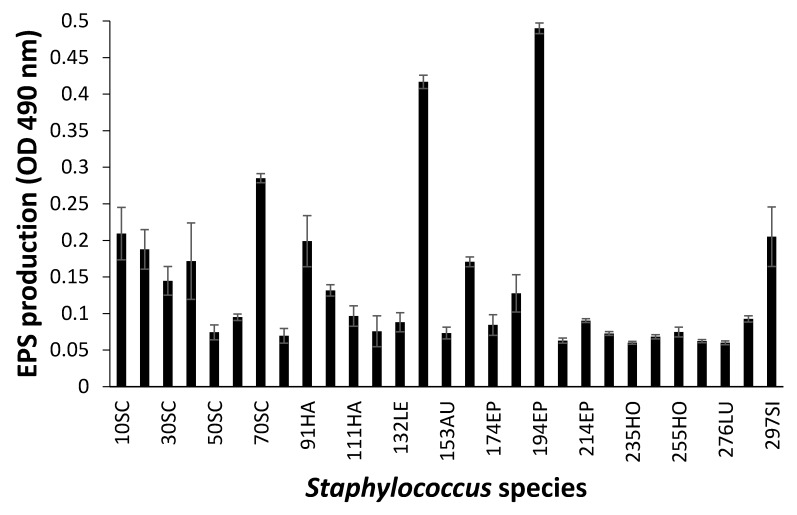
Biofilm formation of *Staphylococcus* species shown by extracellular polymeric substances (EPS) assay. Experiments were run in triplicate and each bar represents the mean ± standard deviation from the mean.

**Figure 4:**
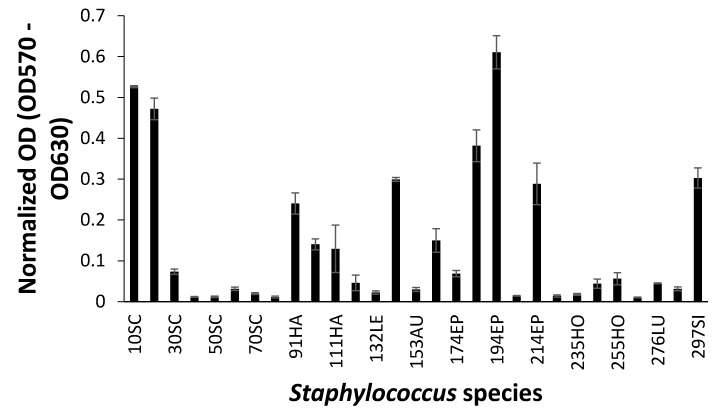
Biofilm formation of *Staphylococcus* species shown by 3-(4,5-dimethyl-2-thiazolyl)-2,5-diphenyl-2H-tetrazolium bromide (MTT) assay. Experiments were run in triplicate and each bar represents the mean ± standard deviation from the mean.

**Figure 5:**
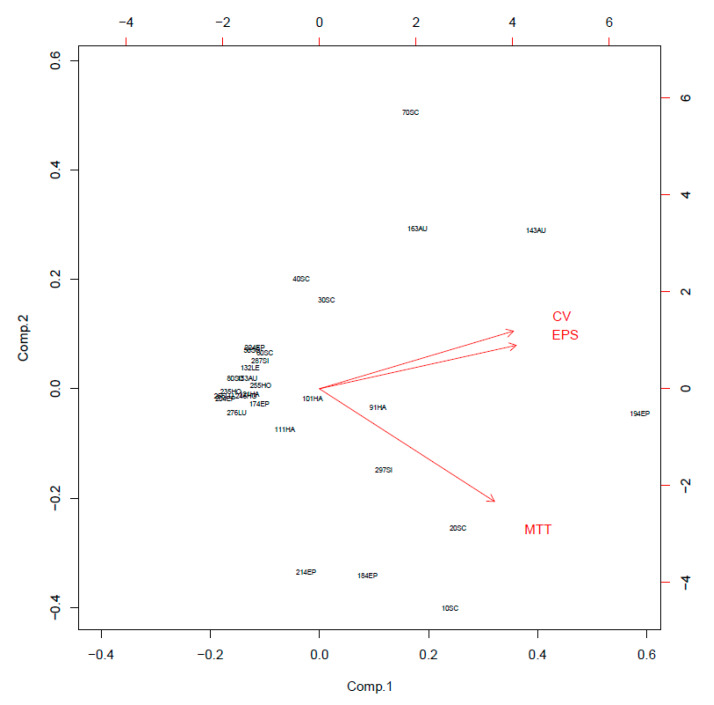
Principal component analysis (PCA) of crystal violet (CV), extracellular polymeric substances (EPS), and 3-(4,5-dimethyl-2-thiazolyl)-2,5-diphenyl-2H-tetrazolium bromide (MTT) assays.

When biofilm was measured by CV staining, CoNS isolates showed a various range of OD_550_ from 0.11 to 0.43 (Figure 2). Seven CoNS strains (OD_550_ > 0.25) formed biofilms very strongly on the polystyrene surfaces. High EPS production (OD_490_ > 0.2) was seen in *S. sciuri* strains 10SC and 70SC, *S. haemolyticus* strain 91HA, *S. auricularis* strains 143AU, *S. epidermidis* strain 194EP and *S. simulans* strain 297SI (Figure 3). The MTT biofilm-formation assay showed that six CoNS strains had higher than 0.30 normalized OD (Figure 4). A three-dimensional space was reduced to a two-dimensional space with Components 1 and 2 by PCA (Figure 5). All quantitative biofilm assays were positively correlated along the Component 1 axis, indicating that higher Component 1 values corresponded to an increase of biofilm formation in CoNS. *S. epidermidis* strain 194EP and *S. auricularis* strains 143AU were described as good biofilm formers by Component 1. Component 2 indicated that EPS and CV methods were inversely correlated with MTT. These observations calculated by PCA were consistent with the results of Spearman rank order correlation coefficients. A high correlation coefficient (ρ = 0.9153, P = 3.612e-12) for CV and EPS was identified (Table 4). Other pairwise comparisons also showed significant correlations between 1.751e-04 and 7.596e-05. None of the correlation coefficients between the prevalence of biofilm-associated genes and biofilm formation assays were significantly different (ρ = −0.01964, 0.06048, and 0.06895 for CV, EPS, and MTT, respectively) (supplementary Table S4).

**Figure 6:**
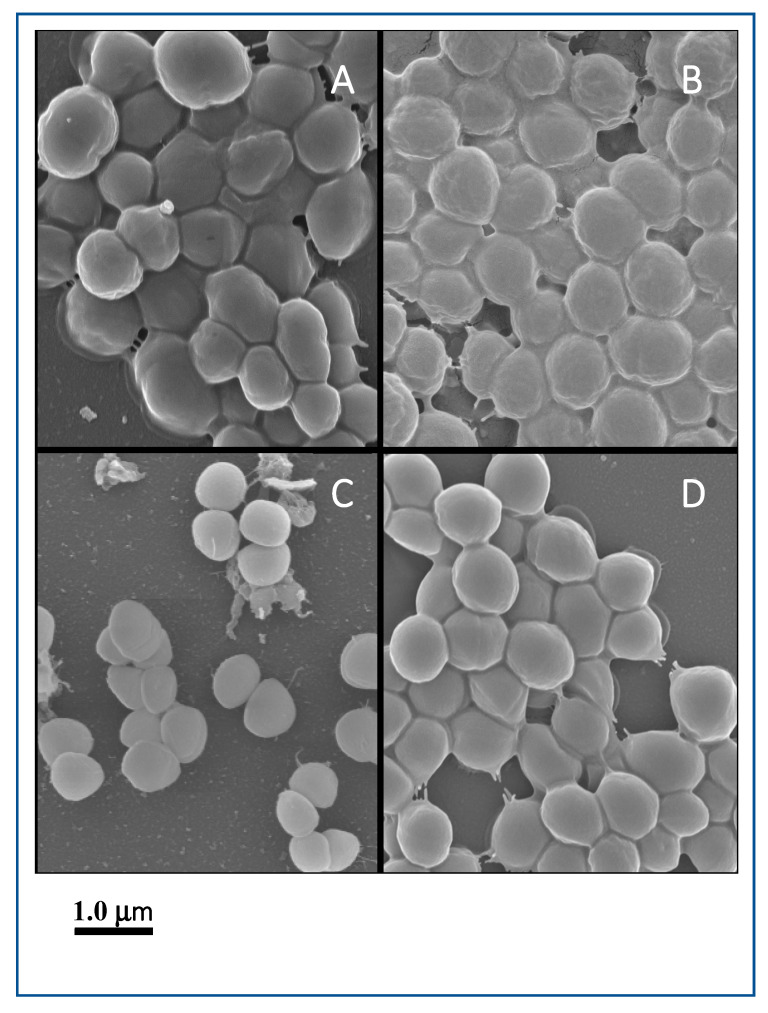
Field emission scanning electron microscopy (FESEM) images of biofilm. The scale bar in all the images corresponds to 1.0 μm. (**A**): *S. sciuri* strain 10SC, (**B**): *S. sciuri* strain 70SC, (**C**): *S. lugdunensis* strain 266LU, (**D**): *S. simulans* strain 297SI.

**Figure 7:**
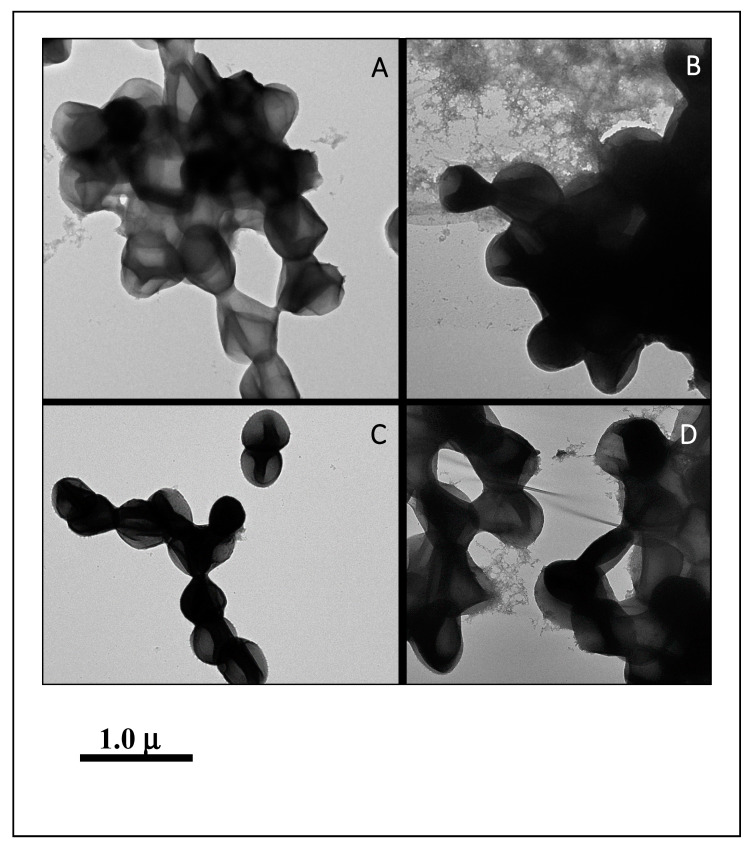
NS-TEM images of biofilm. The scale bar in all the images corresponds to 1.0 μm. (**A**): *S. sciuri* strain 10SC, (**B**): *S. sciuri* strain 70SC, (**C**): *S. lugdunensis* strain 266LU, (**D**): *S. simulans* strain 297SI.

Among good biofilm formers, *S. sciuri* strains 10SC and 70SC were chosen since they had only a single (*eno*) or two (*eno*, *icaA*) biofilm-associated genes. *S. simulans* strain 297SI and *S. lugdunensis* strain 266LU were selected as intermediate and poor biofilm formers. FESEM and NS-TEM analyses of the biofilms in *S. sciuri* strains 10SC and 70SC, *S. lugdunensis* strain 266LU, and *S. simulans* strain 297SI were carried out to examine their architectures (Figure 6 and Figure 7). They had large amounts of extracellular matrix materials or nanofibers extending from the surface that connected bacteria to one another (Figure 6). NS-TEM showed more detailed images of amorphous, loosely adherent extracellular matrix present on the surfaces of the bacterial cells and between cells (Figure 7). Compared to *S. lugdunensis* strain 266LU (Figure 7C), *S. sciuri* strain 70SC showed distinct extracellular components.

## 4. Discussion

In the present study all CoNS isolates demonstrated multidrug-resistance properties, each exhibiting resistance against more than two antimicrobials. Most CoNS were resistant to β-lactam antibiotics and their resistance rates of PEN, OXA and CEF, were 89.7%, 75.9%, and 38.0%, respectively. On the other hand, the resistance rates of VAN and RIF were very low, showing 3.5% and 13.8%, respectively. Although some CoNS (24.1%) were susceptible to OXA, the *mecA* gene was also detected in those isolates. It was reported that staphylococci could exhibit sensitivity to OXA but could harbor *mecA* [45,46,47]. Similarly, we observed that *S. sciuri* strains 40SC, 50SC, 60SC, and *S. epidermidis* strain 224EP carried *aac(6′)-Ie-aph(2′)-Ia*, *aph(3′)-IIIa*, and *aac(6′)-aph(2″), and* they were susceptible to aminoglycoside antibiotics, including gentamicin, kanamycin, and streptomycin. These phenomena have been attributed to the silencing of those resistant genes [48]. Zhu and coworkers also described discordance between genotypic and phenotypic patterns in aminoglycoside resistance in staphylococcal isolates [49].

To better understand the transferability of antibiotic-resistance genes we screened for horizontal transfer genes of conjugative pSK41 and mobilizable pT181 plasmids that are common in staphylococci [50,51]. The presence of megaplasmids and detection of pT181, pSK41, and *traL* genes in some strains indicate that they could carry pT181- or pSK41-type plasmids containing multidrug resistance genes. Recently, several groups have reported that staphylococcal isolates harbor these transfer genes, implying the potential for dissemination of antimicrobial resistance genes to neighboring bacteria [36,52,53]. The presence of genes involved in the transfer of anitimicrobial resistance along with other genes such as DNase, gelatinase, lipase, phosphatase, protease, and urease have been suspected in degrading tissues into nutrients for bacterial growth and contribute to the pathogenicity of CoNS [54].

Among CoNS strains included in this investigation, *S. sciuri* produced five enzymes, including DNase (8/8), gelatinase (5/8), lipase (8/8), phosphatase (8/8), and protease (5/8). These results are in agreement with a previous report indicating the presence of a wide spectrum of virulence factors in *S. sciuri* [38]. We also found the presence of urease, another known virulence factor, in *S. epidermidis*, *S. hominis*, *S. lugdunensis* and *S. simulans.* Urease is normally found in the urinary tract pathogen *S. saprophyticus* [55]. Its presence also been confirmed in other CoNS species, namely *S. capitis* subsp. *ureolyticus*, *S. caprae*, *S. epidermidis*, *S. hominis*, and *S. warneri* [56].

Apart from the virulence factors stated above, the biofilm-forming ability of *S. aureus* and CoNS makes them even more successful pathogens [6,57,58]. It has been shown earlier that *S. epidermidis* has the ability to form biofilms on various biomaterial surfaces and is frequently isolated from patients who suffer from infections of implanted medical devices [59]. Among six *S. epidermidis* isolates used in our study, four isolates were good biofilm formers and *S. epidermidis* species contained seven biofilm-associated genes (*eno*, *fnbB*, *clfA*, *clfB*, *icaA*, *icaB*, *icaD*). One of the *S. epidermidis* strains, particularly 174EP, carried the highest number of biofilm-associated genes. Moreover, several adhesin genes such as *clfA* and *fnbB* in *S. sciuri*, *clfA* and *eno* in *S. lentus*, *clfA* and *fib* in *S. auricularis*, *clfA*, *clfB*, and *fnbB* in *S. epidermidis*, and *clfA* in *S. lugdunensis* were, to the best of our knowledge, the first that we observed in CoNS isolates.

Many different quantitative methods have been used to measure biofilm formation, including CV, EPS, MTT, Congo red agar plates and colony count assays [60]. EPS plays a major role in biofilm formation; their essential components are polysaccharides, lipids, extracellular DNA, metabolites and proteins secreted by bacteria within the biofilm [61]. The amount of EPS produced is considered to be proportional to biofilm formation [62]. The MTT assay has been used to measure respiratory activity of live cells and could be useful to determine the bacterial numbers in biofilm cells [63,64]. Among biofilm formation testing methods, while the CV assay had a good correlation (ρ = 0.9153) with the EPS assay, it had a low correlation (ρ = 0.6418) with the MTT assay. Several studies agreed that there is a close association between biofilm formation estimated by the CV method and EPS production [43,65,66]. Studies that assessed correlation between CV and MTT methods have reported conflicting results. Although some studies found that the CV assay was positively correlated with MTT [67,68,69], other studies did not [70,71,72].

There was no significant relationship between the prevalence of biofilm-associated genes and biofilm formation (supplementary Table S4). For example, although *S. sciuri* strain 60SC had *eno*, *fnbB*, *clfA*, and *icaA* genes, its biofilm-forming ability was very poor compared to the good biofilm formers (*S. sciuri* strains 10SC and 70SC) that harbored *eno* only or *eno* and *icaA*, respectively. Furthermore, the *ica* operon was not associated with biofilm formation. Several researchers documented that multidrug-resistant bacteria were able to form biofilms better than susceptible bacteria [73,74]. However, we found no correlation between antibiotic resistance and biofilm formation. High variability in biofilm formation was found among different species as well as within the same species.

In summary, our study found that the *mecA*-positive CoNS clinical strains possessed a variety of virulence factors, including biofilm formation capability, extracellular enzymes, multidrug resistance and genetic components essential for resistance transfers. Biofilm-forming CoNS strains resistant to many antimicrobials and harboring several virulence enzymes could be of particular concern for critically ill hospitalized patients undergoing antimicrobial therapy, especially when infections associated with medical devices are related to biofilms. Our study highlights a critical need, understanding and continuous monitoring of CoNS-associated nosocomial infections and their trend to elucidate the precise functions of virulence mechanisms and their interactions with host cells under in vivo conditions. We believe that the study could be useful in developing the mitigation strategies to manage and control CoNs infections.

## Figures and Tables

**Table 1:** Phenotypic and genotypic antimicrobial resistance and transfer genes in clinical coagulase-negative staphylococci (CoNS) isolates.

Strain	Species	Source	AMP	BAC	CEF	CIP	ERY	GEN	KAN	LIN	NOV	OXA	PEN	POL	RIF	STR	TET	VAN	Antibiotic Resistance and Transfer Genes
10SC	*sciuri*	Nasal																	*aac(6′)-Ie-aph(2′)-Ia, mecA, **pre_pSK41_***
20SC	*sciuri*	Nasal																	*aac(6′)-Ie-aph(2′)-Ia, mecA*
30SC	*sciuri*	Nasal																	*aac(6′)-Ie-aph(2′)-Ia, mecA*
40SC	*sciuri*	Nasal																	*aac(6′)-Ie-aph(2′)-Ia, mecA*
50SC	*sciuri*	Nasal																	*aph(3′)-IIIa, aac(6′)-Ie-aph(2′)-Ia, ermB, mecA, **pre_pSK41_***
60SC	*sciuri*	Nasal																	*aac(6′)-aph(2″), aph(3′)-IIIa, aac(6′)-Ie-aph(2′)-Ia, sat4, blaZ, mecA, **traL***
70SC	*sciuri*	Nasal																	*aac(6′)-aph(2″), aph(3′)-IIIa, aac(6′)-Ie-aph(2′)-Ia, ermB, sat4, mecA*
80SC	*sciuri*	Nasal																	*aph(3′)-IIIa, aac(6′)-Ie-aph(2′)-Ia, ermB, mecA, **pre_pSK41_**, **pre_pT181_***
91HA	*haemolyticus*	Arterial line catheter																	*aac(6′)-aph(2″), ant(4′)-Ia, aph(3′)-IIIa, aac(6′)-Ie-aph(2′)-Ia, ermB, blaZ, mecA, **pre_pSK41_***
101HA	*haemolyticus*	Nasal																	*aac(6′)-aph(2″), aph(3′)-IIIa, aac(6′)-Ie-aph(2′)-Ia, ermB, sat4, tetL, blaZ, mecA, **pre_pSK41_**, **pre_pT181_***
111HA	*haemolyticus*	Nasal																	*aac(6′)-aph(2″), aph(3′)-IIIa, aac(6′)-Ie-aph(2′)-Ia, ermB, sat4, tetL, blaZ, mecA, **pre_pSK41_**, **pre_pT181_***
121HA	*haemolyticus*	Nasal																	*aac(6′)-aph(2″), aph(3′)-IIIa, aac(6′)-Ie-aph(2′)-Ia, ermB, sat4, mecA, **pre_pT181_**, **traL***
132LE	*lentus*	Nasal																	*aac(6′)-aph(2″), ant(4′)-Ia, aph(3′)-IIIa, aac(6′)-Ie-aph(2′)-Ia, ermB, mecA, **pre_pSK41_**, **pre_pT181_***
143AU	*auricularis*	NA																	*ant(4′)-Ia, aph(3′)-IIIa, aac(6′)-Ie-aph(2′)-Ia, ermB, tetK, blaZ, mecA, **pre_pSK41_**, **pre_pT181_***
153AU	*auricularis*	Blood																	*aac(6′)-aph(2″), aph(3′)-IIIa, aac(6′)-Ie-aph(2′)-Ia, ermB, tetK, blaZ, mecA, **pre_pSK41_**, **pre_pT181_***
163AU	*auricularis*	Blood																	*aac(6′)-aph(2″), aph(3′)-IIIa, aac(6′)-Ie-aph(2′)-Ia, blaZ, mecA, **pre_pT181_***
174EP	*epidermidis*	Blood																	*ermB, mecA, **pre_pT181_***
184EP	*epidermidis*	Wound foot																	*aph(3′)-IIIa, aac(6′)-Ie-aph(2′)-Ia, ermB, sat4, mecA, **pre_pT181_***
194EP	*epidermidis*	Wound buttock																	*tetK, mecA, **pre_pT181_***
204EP	*epidermidis*	NA																	*aac(6′)-aph(2″), ant(4′)-Ia, aac(6′)-Ie-aph(2′)-Ia, mecA, **pre_pSK41_**, **pre_pT181_***
214EP	*epidermidis*	Arterial line catheter																	*aac(6′)-aph(2″), ant(4′)-Ia, aph(3′)-IIIa, aac(6′)-Ie-aph(2′)-Ia, ermB, tetK, blaZ, mecA, **pre_pSK41,_ pre_pT181_***
224EP	*epidermidis*	Perirectal																	*aac(6′)-aph(2″), aac(6′)-Ie-aph(2′)-Ia, ermB, tetK, tetL, mecA, **pre_pSK41,_ pre_pT181_***
235HO	*hominis*	Blood																	*aac(6′)-aph(2″), ant(4′)-Ia, aph(3′)-IIIa, aac(6′)-Ie-aph(2′)-Ia, ermB, mecA, **pre_pSK41,_ pre_pT181_***
245HO	*hominis*	Wound foot																	*aac(6′)-Ie-aph(2′)-Ia, mecA, **pre_pT181_***
255HO	*hominis*	Urine																	*aac(6′)-Ie-aph(2′)-Ia, mecA*
266LU	*lugdunensis*	Wound foot																	*aac(6′)-Ie-aph(2′)-Ia, mecA, **pre_pT181_***
276LU	*lugdunensis*	Arm abscess																	*aph(3′)-IIIa, aac(6′)-Ie-aph(2′)-Ia, ermB, mecA, **pre_pSK41_**, **pre_pT181_***
287SI	*simulans*	Wound thigh																	*aph(3′)-IIIa, aac(6′)-Ie-aph(2′)-Ia, blaZ, mecA, **pre_pT181_***
297SI	*simulans*	Blood																	*aac(6′)-Ie-aph(2′)-Ia, mecA*

* AMP: Ampicillin, BAC: Bacitracin, CEF: Cefazolin, CIP: Ciprofloxacin, ERY: Erythromycin, GEN: Gentamicin, KAN: Kanamycin, LIN: Lincomycin, NOV: Novobiocin, OXA: Oxacillin, PEN: Penicillin, POL: Polymyxin B, RIF: Rifampicin, STR: Streptomycin, TET: Tetracycline, VAN: Vancomycin, ** Solid: Resistant, Grey: Intermediate, Clear: Sensitive. *** Antimicrobial resistance genes are regular and transfer genes are bold; *str, tetM, tetS, tetW*, class I integron, *nes*pSK41, *traE*, *traG*, *traK*, and *traM* were PCR-negative for all CoNS. **** NA: Source is not available.

**Table 2:** Phenotypic virulence enzymes in clinical CoNS isolates.

Strain	COG	DNA	GEL	LIP	PHO	PRO	URE
10SC	−	+	−	+	+	−	−
20SC	−	+	+	+	+	+	−
30SC	−	+	+	+	+	+	−
40SC	−	+	+	+	+	+	−
50SC	−	+	−	+	+	−	−
60SC	−	+	+	+	+	+	−
70SC	−	+	+	+	+	+	−
80SC	−	+	−	+	+	−	−
91HA	−	+	−	−	−	−	−
101HA	−	+	−	−	−	−	−
111HA	−	+	−	−	−	−	−
121HA	−	+	−	−	−	−	−
132LE	−	−	−	+	+	−	−
143AU	−	+	−	+	−	−	−
153AU	−	+	−	+	−	−	−
163AU	−	+	−	+	−	−	−
174EP	−	+	−	+	+	+	+
184EP	−	+	−	−	−	−	+
194EP	−	+	−	+	−	−	+
204EP	−	+	−	+	+	−	+
214EP	−	+	−	+	+	−	+
224EP	−	+	−	−	+	−	+
235HO	−	−	−	−	−	−	+
245HO	−	+	−	+	−	−	−
255HO	−	+	−	−	−	−	+
266LU	−	+	−	+	+	−	+
276LU	−	+	−	+	+	−	+
287SI	−	+	−	+	−	−	+
297SI	−	+	−	−	−	−	+

* COG: Coagulase, DNA: DNase, GEL: Gelatinase, LIP: Lipase, PHO: Phosphatase, URE: Urease, PRO: Protease. ** +: positive, −: negative.

**Table 3:** Genotypic virulence factors in clinical CoNS isolates.

Strain	*eno*	*fnbB*	*fib*	*clfA*	*clfB*	*bap*	*icaA*	*icaB*	*icaD*
10SC	+	−	-	-	−	−	−	−	−
20SC	+	−	−	−	−	−	+	−	−
30SC	+	−	−	−	−	−	+	−	−
40SC	+	−	−	−	−	−	+	−	−
50SC	+	−	−	−	−	−	+	−	−
60SC	+	+	−	+	−	−	+	−	−
70SC	+	−	−	−	−	−	+	−	−
80SC	+	−	−	+	−	−	−	−	−
91HA	+	−	−	−	−	−	−	−	−
101HA	+	−	−	−	−	−	−	−	−
111HA	+	−	−	−	−	−	−	−	−
121HA	+	+	−	−	−	−	+	−	−
132LE	+	−	−	+	−	−	−	−	−
143AU	+	−	−	+	−	+	−	−	−
153AU	+	−	+	+	−	−	−	−	−
163AU	+	−	+	+	−	−	−	−	−
174EP	+	+	−	+	+	−	+	+	+
184EP	+	−	−	+	−	−	−	−	−
194EP	+	−	−	+	−	−	+	−	−
204EP	+	−	−	−	−	−	−	−	−
214EP	+	−	−	+	−	−	+	+	+
224EP	+	−	−	−	−	−	−	−	−
235HO	+	−	−	−	−	−	−	−	−
245HO	+	−	−	−	−	−	−	−	−
255HO	+	−	−	−	−	−	−	−	−
266LU	+	−	−	+	−	−	+	−	−
276LU	+	−	−	+	−	−	+	−	−
287SI	+	−	−	+	−	−	+	−	−
297SI	+	−	−	+	−	−	−	−	−

* All negative results in genotypic tests—cna, ebpS, fnbA, bbp, hla, hlb, sea, seb, sec, sed, see, seg, seh, sei, sej, Tst, eta, etb, pvl.

**Table 4:** Pairwise Spearman rank order correlation coefficient (ρ) for biofilm analysis methods.

	EPS	CV
CV	ρ: 9.153 × 10^−1^ *p* value: 3.612 × 10^−12^	
MTT	ρ: 6.677 × 10^−1^ *p* value: 7.596 × 10^−5^	ρ: 6.418 × 10^−1^ *p* value: 1.751 × 10^−4^

## Supplementary Materials

The following are available online at https://www.mdpi.com/2076-2607/8/5/659/s1.

## Disclaimer

We thank John Sutherland, Jing Han and Youngbeom Ahn for reviewing the manuscript. The views expressed herein do not necessarily reflect those of the US Food and Drug Administration or the US Department of Health and Human Services. The mention of commercial products, their sources, or their use in connection with material reported herein is not to be construed as either an actual or implied endorsement of such products by the Department of Health and Human Services. The findings and conclusions in this Perspective have not been formally disseminated by the US Food and Drug Administration and should not be construed to represent any agency determination or policy.

## References

[1] Becker K., Heilmann C., Peters G. (2014). Coagulase-negative staphylococci. Clin. Microbiol. Rev..

[2] Couto I., Pereira S., Miragaia M., Sanches I.S., de Lencastre H. (2001). Identification of clinical staphylococcal isolates from humans by internal transcribed spacer PCR. J. Clin. Microbiol..

[3] Piette A., Verschraegen G. (2009). Role of coagulase-negative staphylococci in human disease. Vet. Microbiol..

[4] Szczuka E., Jablonska L., Kaznowski A. (2016). Coagulase-negative staphylococci: Pathogenesis, occurrence of antibiotic resistance genes and in vitro effects of antimicrobial agents on biofilm-growing bacteria. J. Med. Microbiol..

[5] Vautor E., Abadie G., Pont A., Thiery R. (2008). Evaluation of the presence of the bap gene in Staphylococcus aureus isolates recovered from human and animals species. Vet. Microbiol..

[6] Atshan S.S., Nor Shamsudin M., Sekawi Z., Lung L.T., Hamat R.A., Karunanidhi A., Mateg Ali A., Ghaznavi-Rad E., Ghasemzadeh-Moghaddam H., Chong Seng J.S. (2012). Prevalence of adhesion and regulation of biofilm-related genes in different clones of Staphylococcus aureus. J. Biomed. Biotechnol..

[7] Vogel L., Sloos J.H., Spaargaren J., Suiker I., Dijkshoorn L. (2000). Biofilm production by Staphylococcus epidermidis isolates associated with catheter related bacteremia. Diagn. Microbiol. Infect. Dis..

[8] Cerca N., Martins S., Cerca F., Jefferson K.K., Pier G.B., Oliveira R., Azeredo J. (2005). Comparative assessment of antibiotic susceptibility of coagulase-negative staphylococci in biofilm versus planktonic culture as assessed by bacterial enumeration or rapid XTT colorimetry. J. Antimicrob. Chemother..

[9] Beceiro A., Tomas M., Bou G. (2013). Antimicrobial resistance and virulence: A successful or deleterious association in the bacterial world?. Clin. Microbiol. Rev..

[10] Dakic I., Morrison D., Vukovic D., Savic B., Shittu A., Jezek P., Hauschild T., Stepanovic S. (2005). Isolation and molecular characterization of Staphylococcus sciuri in the hospital environment. J. Clin. Microbiol..

[11] Silva F.R., Mattos E.M., Coimbra M.V., Ferreira-Carvalho B.T., Figueiredo A.M. (2001). Isolation and molecular characterization of methicillin-resistant coagulase-negative staphylococci from nasal flora of healthy humans at three community institutions in Rio de Janeiro City. Epidemiol. Infect..

[12] Unal N., Cinar O.D. (2012). Detection of stapylococcal enterotoxin, methicillin-resistant and Panton-Valentine leukocidin genes in coagulase-negative staphylococci isolated from cows and ewes with subclinical mastitis. Trop. Anim. Health Prod..

[13] Barbier F., Ruppe E., Hernandez D., Lebeaux D., Francois P., Felix B., Desprez A., Maiga A., Woerther P.L., Gaillard K. (2010). Methicillin-resistant coagulase-negative staphylococci in the community: High homology of SCCmec IVa between Staphylococcus epidermidis and major clones of methicillin-resistant Staphylococcus aureus. J. Infect. Dis..

[14] Chong J., Caya C., Levesque S., Quach C. (2016). Heteroresistant Vancomycin Intermediate Coagulase Negative Staphylococcus in the NICU: A Systematic Review. PLoS ONE.

[15] Brinkmann V., Reichard U., Goosmann C., Fauler B., Uhlemann Y., Weiss D.S., Weinrauch Y., Zychlinsky A. (2004). Neutrophil extracellular traps kill bacteria. Science.

[16] Rosenstein R., Gotz F. (2000). Staphylococcal lipases: Biochemical and molecular characterization. Biochimie.

[17] Dubin G. (2002). Extracellular proteases of *Staphylococcus* spp.. Biol. Chem..

[18] Potempa J., Pike R.N. (2009). Corruption of innate immunity by bacterial proteases. J. Innate Immun..

[19] Pizauro L.J.L., de Almeida C.C., Soltes G.A., Slavic D., de Avila F.A., Zafalon L.F., MacInnes J.I. (2019). Short communication: Detection of antibiotic resistance, mecA, and virulence genes in coagulase-negative Staphylococcus spp. from buffalo milk and the milking environment. J. Dairy Sci..

[20] Ortega-Pena S., Franco-Cendejas R., Salazar-Saenz B., Rodriguez-Martinez S., Cancino-Diaz M.E., Cancino-Diaz J.C. (2019). Prevalence and virulence factors of coagulase negative Staphylococcus causative of prosthetic joint infections in an orthopedic hospital of Mexico. Cir. Cir..

[21] Shakir Z., Khan S., Sung K., Khare S., Khan A., Steele R., Nawaz M. (2012). Molecular characterization of fluoroquinolone-resistant *Aeromonas* spp. isolated from imported shrimp. Appl. Environ. Microbiol..

[22] CLSI (2015). Performance Standards for Antimicrobial Susceptibility Testing. 25th Informational Supplement.

[23] Coia J.E., Noor-Hussain I., Platt D.J. (1988). Plasmid profiles and restriction enzyme fragmentation patterns of plasmids of methicillin-sensitive and methicillin-resistant isolates of Staphylococcus aureus from hospital and the community. J. Med. Microbiol..

[24] Mitchell G., Lafrance M., Boulanger S., Seguin D.L., Guay I., Gattuso M., Marsault E., Bouarab K., Malouin F. (2012). Tomatidine acts in synergy with aminoglycoside antibiotics against multiresistant Staphylococcus aureus and prevents virulence gene expression. J. Antimicrob. Chemother..

[25] Klingenberg C., Sundsfjord A., Ronnestad A., Mikalsen J., Gaustad P., Flaegstad T. (2004). Phenotypic and genotypic aminoglycoside resistance in blood culture isolates of coagulase-negative staphylococci from a single neonatal intensive care unit, 1989–2000. J. Antimicrob. Chemother..

[26] Schnellmann C., Gerber V., Rossano A., Jaquier V., Panchaud Y., Doherr M.G., Thomann A., Straub R., Perreten V. (2006). Presence of new mecA and mph(C) variants conferring antibiotic resistance in *Staphylococcus* spp. isolated from the skin of horses before and after clinic admission. J. Clin. Microbiol..

[27] Rosato A.E., Kreiswirth B.N., Craig W.A., Eisner W., Climo M.W., Archer G.L. (2003). mecA-blaZ corepressors in clinical Staphylococcus aureus isolates. Antimicrob. Agents Chemother..

[28] Nawaz M.S., Khan S.A., Khan A.A., Khambaty F.M., Cerniglia C.E. (2000). Comparative molecular analysis of erythromycin-resistance determinants in staphylococcal isolates of poultry and human origin. Mol. Cell. Probes.

[29] You Y., Hilpert M., Ward M.J. (2012). Detection of a common and persistent tet(L)-carrying plasmid in chicken-waste-impacted farm soil. Appl. Environ. Microbiol..

[30] Trzcinski K., Cooper B.S., Hryniewicz W., Dowson C.G. (2000). Expression of resistance to tetracyclines in strains of methicillin-resistant Staphylococcus aureus. J. Antimicrob. Chemother..

[31] Aminov R.I., Garrigues-Jeanjean N., Mackie R.I. (2001). Molecular ecology of tetracycline resistance: Development and validation of primers for detection of tetracycline resistance genes encoding ribosomal protection proteins. Appl. Environ. Microbiol..

[32] Ren C., Zhao Y., Shen Y. (2013). Analysis of the effect of integrons on drug-resistant Staphylococcus aureus by multiplex PCR detection. Mol. Med. Rep..

[33] Seo Y.S., Lee D.Y., Rayamahji N., Kang M.L., Yoo H.S. (2008). Biofilm-forming associated genotypic and phenotypic characteristics of *Staphylococcus* spp. isolated from animals and air. Res. Vet. Sci..

[34] Tormo M.A., Knecht E., Gotz F., Lasa I., Penades J.R. (2005). Bap-dependent biofilm formation by pathogenic species of Staphylococcus: Evidence of horizontal gene transfer?. Microbiology.

[35] Park J.Y., Fox L.K., Seo K.S., McGuire M.A., Park Y.H., Rurangirwa F.R., Sischo W.M., Bohach G.A. (2011). Detection of classical and newly described staphylococcal superantigen genes in coagulase-negative staphylococci isolated from bovine intramammary infections. Vet. Microbiol..

[36] Aguila-Arcos S., Alvarez-Rodriguez I., Garaiyurrebaso O., Garbisu C., Grohmann E., Alkorta I. (2017). Biofilm-Forming Clinical Staphylococcus Isolates Harbor Horizontal Transfer and Antibiotic Resistance Genes. Front. Microbiol..

[37] Garcia M.L., Moreno B., Bergdoll M.S. (1980). Characterization of staphylococci isolated from mastitic cows in Spain. Appl. Environ. Microbiol..

[38] Stepanovic S., Vukovicc D., Trajkovic V., Samardzic T., Cupic M., Svabic-Vlahovic M. (2001). Possible virulence factors of Staphylococcus sciuri. FEMS Microbiol. Lett..

[39] Chakraborty S.P., Mahapatra S.K., Roy S. (2011). Biochemical characters and antibiotic susceptibility of Staphylococcus aureus isolates. Asian Pac. J. Trop. Biomed..

[40] Reynolds H.T., Barton H.A. (2014). Comparison of the white-nose syndrome agent Pseudogymnoascus destructans to cave-dwelling relatives suggests reduced saprotrophic enzyme activity. PLoS ONE.

[41] Coffey B.M., Anderson G.G. (2014). Biofilm formation in the 96-well microtiter plate. Methods Mol. Biol..

[42] Cady N.C., McKean K.A., Behnke J., Kubec R., Mosier A.P., Kasper S.H., Burz D.S., Musah R.A. (2012). Inhibition of biofilm formation, quorum sensing and infection in Pseudomonas aeruginosa by natural products-inspired organosulfur compounds. PLoS ONE.

[43] Kulshrestha S., Khan S., Hasan S., Khan M.E., Misba L., Khan A.U. (2016). Calcium fluoride nanoparticles induced suppression of Streptococcus mutans biofilm: An in vitro and in vivo approach. Appl. Microbiol. Biotechnol..

[44] Xu J., Chen Y., Zhang R., Song Y., Cao J., Bi N., Wang J., He J., Bai J., Dong L. (2013). Global and targeted metabolomics of esophageal squamous cell carcinoma discovers potential diagnostic and therapeutic biomarkers. Mol. Cell. Proteom..

[45] Ikonomidis A., Michail G., Vasdeki A., Labrou M., Karavasilis V., Stathopoulos C., Maniatis A.N., Pournaras S. (2008). In vitro and in vivo evaluations of oxacillin efficiency against mecA-positive oxacillin-susceptible Staphylococcus aureus. Antimicrob. Agents Chemother..

[46] Kumar V.A., Steffy K., Chatterjee M., Sugumar M., Dinesh K.R., Manoharan A., Karim S., Biswas R. (2013). Detection of oxacillin-susceptible mecA-positive Staphylococcus aureus isolates by use of chromogenic medium MRSA ID. J. Clin. Microbiol..

[47] Hososaka Y., Hanaki H., Endo H., Suzuki Y., Nagasawa Z., Otsuka Y., Nakae T., Sunakawa K. (2007). Characterization of oxacillin-susceptible mecA-positive Staphylococcus aureus: A new type of MRSA. J. Infect. Chemother..

[48] Enne V.I., Delsol A.A., Roe J.M., Bennett P.M. (2006). Evidence of antibiotic resistance gene silencing in Escherichia coli. Antimicrob. Agents Chemother..

[49] Zhu L.X., Zhang Z.W., Wang C., Yang H.W., Jiang D., Zhang Q., Mitchelson K., Cheng J. (2007). Use of a DNA microarray for simultaneous detection of antibiotic resistance genes among staphylococcal clinical isolates. J. Clin. Microbiol..

[50] Berg T., Firth N., Apisiridej S., Hettiaratchi A., Leelaporn A., Skurray R.A. (1998). Complete nucleotide sequence of pSK41: Evolution of staphylococcal conjugative multiresistance plasmids. J. Bacteriol..

[51] Novick R.P. (1989). Staphylococcal plasmids and their replication. Annu. Rev. Microbiol..

[52] Schiwon K., Arends K., Rogowski K.M., Furch S., Prescha K., Sakinc T., Van Houdt R., Werner G., Grohmann E. (2013). Comparison of antibiotic resistance, biofilm formation and conjugative transfer of Staphylococcus and Enterococcus isolates from International Space Station and Antarctic Research Station Concordia. Microb. Ecol..

[53] Koning S., van Belkum A., Snijders S., van Leeuwen W., Verbrugh H., Nouwen J., Op’t Veld M., van Suijlekom-Smit L.W., van der Wouden J.C., Verduin C. (2003). Severity of nonbullous Staphylococcus aureus impetigo in children is associated with strains harboring genetic markers for exfoliative toxin B, Panton-Valentine leukocidin, and the multidrug resistance plasmid pSK41. J. Clin. Microbiol..

[54] Dinges M.M., Orwin P.M., Schlievert P.M. (2000). Exotoxins of Staphylococcus aureus. Clin. Microbiol. Rev..

[55] Loes A.N., Ruyle L., Arvizu M., Gresko K.E., Wilson A.L., Deutch C.E. (2014). Inhibition of urease activity in the urinary tract pathogen Staphylococcus saprophyticus. Lett. Appl. Microbiol..

[56] Bannerman T.L., Kloos W.E. (1991). Staphylococcus capitis subsp. ureolyticus subsp. nov. from human skin. Int. J. Syst. Bacteriol..

[57] Khoramian B., Jabalameli F., Niasari-Naslaji A., Taherikalani M., Emaneini M. (2015). Comparison of virulence factors and biofilm formation among Staphylococcus aureus strains isolated from human and bovine infections. Microb. Pathog..

[58] Simojoki H., Hyvonen P., Plumed Ferrer C., Taponen S., Pyorala S. (2012). Is the biofilm formation and slime producing ability of coagulase-negative staphylococci associated with the persistence and severity of intramammary infection?. Vet. Microbiol..

[59] Mack D., Davies A.P., Harris L.G., Rohde H., Horstkotte M.A., Knobloch J.K. (2007). Microbial interactions in Staphylococcus epidermidis biofilms. Anal. Bioanal. Chem..

[60] Azeredo J., Azevedo N.F., Briandet R., Cerca N., Coenye T., Costa A.R., Desvaux M., Di Bonaventura G., Hebraud M., Jaglic Z. (2017). Critical review on biofilm methods. Crit. Rev. Microbiol..

[61] Di Martino P. (2018). Extracellular polymeric substances, a key element in understanding biofilm phenotype. AIMS Microbiol..

[62] Stiefel P., Rosenberg U., Schneider J., Mauerhofer S., Maniura-Weber K., Ren Q. (2016). Is biofilm removal properly assessed? Comparison of different quantification methods in a 96-well plate system. Appl. Microbiol. Biotechnol..

[63] Trafny E.A., Lewandowski R., Zawistowska-Marciniak I., Stepinska M. (2013). Use of MTT assay for determination of the biofilm formation capacity of microorganisms in metalworking fluids. World J. Microbiol. Biotechnol..

[64] Traba C., Liang J.F. (2011). Susceptibility of Staphylococcus aureus biofilms to reactive discharge gases. Biofouling.

[65] Silva-Dias A., Miranda I.M., Branco J., Monteiro-Soares M., Pina-Vaz C., Rodrigues A.G. (2015). Adhesion, biofilm formation, cell surface hydrophobicity, and antifungal planktonic susceptibility: Relationship among *Candida* spp.. Front. Microbiol..

[66] Ochoa S.A., Cruz-Cordova A., Rodea G.E., Cazares-Dominguez V., Escalona G., Arellano-Galindo J., Hernandez-Castro R., Reyes-Lopez A., Xicohtencatl-Cortes J. (2015). Phenotypic characterization of multidrug-resistant Pseudomonas aeruginosa strains isolated from pediatric patients associated to biofilm formation. Microbiol. Res..

[67] Li X., Yan Z., Xu J. (2003). Quantitative variation of biofilms among strains in natural populations of Candida albicans. Microbiology.

[68] Raut J.S., Shinde R.B., Chauhan N.M., Karuppayil S.M. (2014). Phenylpropanoids of plant origin as inhibitors of biofilm formation by Candida albicans. J. Microbiol. Biotechnol..

[69] Weerasekera M.M., Wijesinghe G.K., Jayarathna T.A., Gunasekara C.P., Fernando N., Kottegoda N., Samaranayake L.P. (2016). Culture media profoundly affect Candida albicans and Candida tropicalis growth, adhesion and biofilm development. Mem. Inst. Oswaldo Cruz..

[70] Jin Y., Yip H.K., Samaranayake Y.H., Yau J.Y., Samaranayake L.P. (2003). Biofilm-forming ability of Candida albicans is unlikely to contribute to high levels of oral yeast carriage in cases of human immunodeficiency virus infection. J. Clin. Microbiol..

[71] Premamalini T., Anitha S., Mohanapriya K., Kindo A.J. (2018). Evaluation of 3-(4,5-dimethylthiazol-2-yl)-2,5-diphenyl tetrazolium bromide method for assessing biofilm formation in vitro by *Trichosporon* spp.. J. Lab. Physicians.

[72] Melo A.S., Bizerra F.C., Freymuller E., Arthington-Skaggs B.A., Colombo A.L. (2011). Biofilm production and evaluation of antifungal susceptibility amongst clinical Candida spp. isolates, including strains of the Candida parapsilosis complex. Med. Mycol..

[73] Abidi S.H., Sherwani S.K., Siddiqui T.R., Bashir A., Kazmi S.U. (2013). Drug resistance profile and biofilm forming potential of Pseudomonas aeruginosa isolated from contact lenses in Karachi-Pakistan. BMC Ophthalmol..

[74] Gurung J., Khyriem A.B., Banik A., Lyngdoh W.V., Choudhury B., Bhattacharyya P. (2013). Association of biofilm production with multidrug resistance among clinical isolates of Acinetobacter baumannii and Pseudomonas aeruginosa from intensive care unit. Indian J. Crit. Care Med..

