# Supplementary material for: Virulence Characteristics of mecA-Positive Multidrug-Resistant Clinical Coagulase-Negative Staphylococci
Source: Microorganisms. 2020 May 1;8(5):659. doi: 10.3390/microorganisms8050659 (PMC7284987; doi:10.3390/microorganisms8050659)
Supplement: Supplementary file 1 [file microorganisms-08-00659-s001.pdf]

Supplemental materials

Table 1. Oligonucleotides used for amplification of antibiotic-resistant genes.

Primers	Nucleotide Sequence (5'-3')	Reference
aac(6')-aph(2")_For	ACAGAGCTTGGGAAGATGAAGT	[1]
aac(6')-aph(2")_Rev	GCCCTCGTGTAATTCATGTTCTGGC	
ant(4')-Ia_For	CTGCTAAATCGGTAGAAGC	[1]
ant(4')-Ia_Rev	CAGACCAATCAACATGGCACC	
aph(3')-IIIa_For	CTGATCGAAAAATACCGCTGC	[1]
aph(3')-IIIa_Rev	TCATACTCTTCCGAGCAAAGG	
aac(6')-Ie-aph(2')-Ia_For	CAGAGCCTTGGGAAGATGAAG	[1]
aac(6')-Ie-aph(2')-Ia_Rev	CCTCGTGTAATTCATGTTCTGGC	
blaZ_For	TCAAACAGTTCACATGCC	[2]
blaZ_Rev	TTCATTACACTCTGGCG	
Class I integron_For	CCTCCCGCACGATGATC	[3]
Class I integron_Rev	TCCACGCATCGTCAGGC	
ermB_For	TAACGACGAAACTGGCTAAAAT	[4]
ermB_Rev	ATCTGTGGTATGGCGGGTAAG	
mecA_For	ATGAAAAAGATAAAAATTGTTCCAC	[5]
mecA_Rev	TTATTCATCTATATCGTATTTTTTATTAC	
sat4_For	CGATAAACCCAGCGAACC	[5]
sat4_Rev	ATAACATAGTATCGACGG	
str_For	TATTGCTCTCGAGGGTTC	[1]
str_Rev	CTTTCTATATCCATTCATCTC	
tetK_For	TTAGGTGAAGGGTTAGGTCC	[6]
tetK_Rev	GCAAACTCATTCCAGAAGCA	
tetL_For	ATAAATTGTTTCGGGTCGGTAAT	[7]
tetL_Rev	AACCAGCCAACTAATGACAATGAT	
tetM_For	ACAGAAAGCTTATTATATAAC	[8]
tetM_Rev	TGGCGTGTCTATGATGTTCAC	
tetS_For	GAAAGCTTACTATACAGTAGC	[8]
tetS_Rev	AGGAGTATCTACAATATTTAC	
tetW_For	GAGAGCCTGCTATATGCCAGC	[8]
tetW_Rev	GGGCGTATCCACAATGTTAAC	

Table 2. Oligonucleotides used for amplification of horizontal transfer genes.

Primers	Nucleotide Sequence (5'-3')	Reference
pre_{pSK41}_For	CTGGACTAAAAGGCATGCAA	[9]
pre_{pSK41}_Rev	GCAGTTTTCCATCACGCATA	
nes_{pSK41}_For	AGCGCTAGTAGGATTAAAG	[9]
nes_{pSK41}_Rev	CATAATAAATGTGCGTGAGG	
pre_{pT181}_For	TCGAACAGAATTATACAGGCAA	[9]
pre_{pT181}_Rev	CTGACTTATTTGCTCATGTTTAGC	
traE_For	TATCATTGATCC(T/C)GAA(A/G)ATGAAT	[9]
traE_Rev	TCTTTTGT(T/G)ATTTCGTCCCATAA	
traG_For	GTGTTGACGGTTCGGGTATC	[9]
traG_Rev	TTTTCCGTCTGAACCTCCAC	
traK_For	TATCTAAAGACCACCCAGCTAGAG	[9]
traK_Rev	TACTTGTTTCAAACTCTACAGTAGC	
traL_For	ATGGGGACTATGGCAGGTAG	[9]
traL_Rev	AAGTTTTGCACCACTTCCAG	
traM_For	TGTTGTATGGGGAAAACAAGC	[9]
traM_Rev	GCTGGGCTTATAGC(A/G)TCATC	

Table 3. Oligonucleotides used for amplification of virulence-associated genes.

Primers	Nucleotide Sequence (5'-3')	Reference
bap_For	CCCTATATCGAAGGTGTAGAATTGCAC	[10]
bap_Rev	GCTGTTGAAGTTAATACTGTACCTGC	
bbp_For	AACTACATCTAGTACTCAACAACAG	[11]
bbp_Rev	ATGTGCTTGAATAACACCATCATCT	
clfA_For	ATTGGCGTGGCTTCAGTGCT	[11]
clfA_Rev	CGTTTCTTCCGTAGTTGCATTTG	
clfB_For	ACATCAGTAATAGTAGGGGGCAAC	[11]
clfB_Rev	TTCGCACTGTTTGTGTTTGCAC	
cna_For	GTCAAGCAGTTATTAACACCAGAC	[11]
cna_Rev	AATCAGTAATTGCACTTTGTCCACTG	
eno_For	ACGTGCAGCAGCTGACT	[11]
eno_Rev	CAACAGCATYCTTCAGTACCTTC	
ebpS_For	CATCCAGAACCAATCGAAGAC	[11]
ebpS_Rev	CTTAACAGTTACATCATCATGTTTATCTTTG	
eta_For	CTAGTGCATTTGTTATTCAA	[12]
eta_Rev	TGCATTGACACCATAGTACT	
etb_For	ACGGCTATATACATTCAATT	[12]
etb_Rev	TCCATCGATAATATACCTAA	
fib_For	CTACAACTACAATTGCCGTCAACAG	[11]
fib_Rev	GCTCTTGTAAGACCATTTTCTTCAC	
fnbA_For	GTGAAGTTTTAGAAGGTGGAAAGATTAG	[11]
fnbA_Rev	GCTCTTGTAAGACCATTTTTCTTCAC	
fnbB_For	GTAACAGCTAATGGTCGAATTGATACT	[11]
fnbB_Rev	CAAGTTCGATAGGAGTACTATGTTC	
icaA_For	TGGGATACTGAYATGATTAC	[13]
icaA_Rev	CCTCTGTCTGGGCTTGACCATG	
icaB_For	CTGATCAAGAATTTAAATCACAAA	[14]
icaB_Rev	AAAGTCCCATAAGCCTGTTT	
icaD_For	AAACGTAAGAGAGGTGG	[15]
icaD_Rev	GGCAATATGATCAAGATAC	
pvl_For	ATCATTAGGTAAAATGTCTGGACATGATCCA	[16]
pvl_Rev	GCATCAASTGTATTGGATAGCAAAAGC	
sea_For	GCAGGGAACAGCTTTAGGC	[17]
sea_Rev	GTTCTGTAGAAGTATGAAACACG	
seb_For	ACATGTAATTTTGATATTCGCACTG	[18]
seb_Rev	TGCAGGCATCATGTCATACCA	
sec_For	CTTGTATGTATGGAGGAATAACAA	[17]
sec_Rev	TGCAGGCATCATATCATACCA	

sed_For	GTGGTGAAATAGATAGGACTGC	[17]
sed_Rev	ATATGAAGGTGCTCTGTGG	
see_For	TACCAATTAACTTGTGGATAGAC	[17]
see_Rev	CTCTTTGCACCTTACCGC	
seg_For	CGTCTCCACCTGTTGAAGG	[17]
seg_Rev	CCAAGTGATTGTCTATTGTCG	
seh_For	CAACTGCTGATTTAGCTCAG	[17]
seh_Rev	GTCGAATGAGTAATCTCTAGG	
sei_For	CAACTCGAATTTTCAACAGGTACC	[17]
sei_Rev	CAGGCAGTCCATCTCCTG	
sej_For	CATCAGAACTGTTGTTCCGCTAG	[17]
sej_Rev	CTGAATTTTACCATCAAAGGTAC	
Tst_For	GCTTGCGACAACTGCTACAG	[18]
Tst_Rev	TGGATCCGTCATTCATTGTTAT	

Table 4. Spearman rank order correlation coefficient (ρ) for biofilm analysis methods and number of biofilm-associated genes.

	**Number of Biofilm-Associated Genes**
CV	ρ: -1.964×10^{-2}<br>p value: 9.194×10^{-1}
EPS	ρ: 6.048×10^{-2}<br>p value: 7.553×10^{-1}
MTT	ρ: 6.895×10^{-2}<br>p value: 7.223×10^{-1}

References

1. Mitchell, G.; Lafrance, M.; Boulanger, S.; Seguin, D.L.; Guay, I.; Gattuso, M.; Marsault, E.; Bouarab, K.; Malouin, F. Tomatidine acts in synergy with aminoglycoside antibiotics against multiresistant Staphylococcus aureus and prevents virulence gene expression. *J. Antimicrob. Chemother.* **2012**, *67*, 559–568, doi:10.1093/jac/dkr510.
2. Rosato, A.E.; Kreiswirth, B.N.; Craig, W.A.; Eisner, W.; Climo, M.W.; Archer, G.L. mecA-blaZ corepressors in clinical Staphylococcus aureus isolates. *Antimicrob. Agents Chemother.* **2003**, *47*, 1460–1463.
3. Ren, C.; Zhao, Y.; Shen, Y. Analysis of the effect of integrons on drug-resistant Staphylococcus aureus by multiplex PCR detection. *Mol. Med. Rep.* **2013**, *7*, 719–724, doi:10.3892/mmr.2013.1284.
4. Nawaz, M.S.; Khan, S.A.; Khan, A.A.; Khambaty, F.M.; Cerniglia, C.E. Comparative molecular analysis of erythromycin-resistance determinants in staphylococcal isolates of poultry and human origin. *Mol. Cell. Probes.* **2000**, *14*, 311–319, doi:10.1006/mcpr.2000.0320.
5. Schnellmann, C.; Gerber, V.; Rossano, A.; Jaquier, V.; Panchaud, Y.; Doherr, M.G.; Thomann, A.; Straub, R.; Perreten, V. Presence of new mecA and mph(C) variants conferring antibiotic resistance in Staphylococcus spp. isolated from the skin of horses before and after clinic admission. *J. Clin. Microbiol.* **2006**, *44*, 4444–4454, doi:10.1128/JCM.00868-06.
6. You, Y.; Hilpert, M.; Ward, M.J. Detection of a common and persistent tet(L)-carrying plasmid in chicken-waste-impacted farm soil. *Appl. Environ. Microbiol.* **2012**, *78*, 3203–3213, doi:10.1128/AEM.07763-11.
7. Trzcinski, K.; Cooper, B.S.; Hryniewicz, W.; Dowson, C.G. Expression of resistance to tetracyclines in strains of methicillin-resistant Staphylococcus aureus. *J. Antimicrob. Chemother.* **2000**, *45*, 763–770.
8. Aminov, R.I.; Garrigues-Jeanjean, N.; Mackie, R.I. Molecular ecology of tetracycline resistance: Development and validation of primers for detection of tetracycline resistance genes encoding ribosomal protection proteins. *Appl. Environ. Microbiol.* **2001**, *67*, 22–32, doi:10.1128/AEM.67.1.22-32.2001.
9. Aguila-Arcos, S.; Alvarez-Rodriguez, I.; Garaiyurrebaso, O.; Garbisu, C.; Grohmann, E.; Alkorta, I. Biofilm-Forming Clinical Staphylococcus Isolates Harbor Horizontal Transfer and Antibiotic Resistance Genes. *Front. Microbiol.* **2017**, *8*, 2018, doi:10.3389/fmicb.2017.02018.
10. Tormo, M.A.; Knecht, E.; Gotz, F.; Lasa, I.; Penades, J.R. Bap-dependent biofilm formation by pathogenic species of Staphylococcus: Evidence of horizontal gene transfer? *Microbiology* **2005**, *151*, 2465–2475, doi:10.1099/mic.0.27865-0.
11. Seo, Y.S.; Lee, D.Y.; Rayamahji, N.; Kang, M.L.; Yoo, H.S. Biofilm-forming associated genotypic and phenotypic characteristics of Staphylococcus spp. isolated from animals and air. *Res. Vet. Sci.* **2008**, *85*, 433–438, doi:10.1016/j.rvsc.2008.01.005.
12. Johnson, W.M.; Tyler, S.D.; Ewan, E.P.; Ashton, F.E.; Pollard, D.R.; Rozee, K.R. Detection of genes for enterotoxins, exfoliative toxins, and toxic shock syndrome toxin 1 in Staphylococcus aureus by the polymerase chain reaction. *J. Clin. Microbiol.* **1991**, *29*, 426–430.
13. Moretro, T.; Hermansen, L.; Holck, A.L.; Sidhu, M.S.; Rudi, K.; Langsrud, S. Biofilm formation and the presence of the intercellular adhesion locus ica among staphylococci from food and food processing environments. *Appl. Environ. Microbiol.* **2003**, *69*, 5648–5655.
14. Solati, S.M.; Tajbakhsh, E.; Khamesipour, F.; Gugnani, H.C. Prevalence of virulence genes of biofilm producing strains of Staphylococcus epidermidis isolated from clinical samples in Iran. *AMB Express* **2015**, *5*, 134, doi:10.1186/s13568-015-0134-3.
15. Vasudevan, P.; Nair, M.K.; Annamalai, T.; Venkitanarayanan, K.S. Phenotypic and genotypic characterization of bovine mastitis isolates of Staphylococcus aureus for biofilm formation. *Vet. Microbiol.* **2003**, *92*, 179–185.
16. Lina, G.; Piemont, Y.; Godail-Gamot, F.; Bes, M.; Peter, M.O.; Gauduchon, V.; Vandenesch, F.; Etienne, J. Involvement of Panton-Valentine leukocidin-producing Staphylococcus aureus in primary skin infections and pneumonia. *Clin. Infect. Dis.* **1999**, *29*, 1128–1132, doi:10.1086/313461.
17. Monday, S.R.; Bohach, G.A. Use of multiplex PCR to detect classical and newly described pyrogenic toxin genes in staphylococcal isolates. *J. Clin. Microbiol.* **1999**, *37*, 3411–3414.
18. Lovseth, A.; Loncarevic, S.; Berdal, K.G. Modified multiplex PCR method for detection of pyrogenic exotoxin genes in staphylococcal isolates. *J. Clin. Microbiol.* **2004**, *42*, 3869–3872, doi:10.1128/JCM.42.8.3869-3872.2004.
